# General practitioners’ type of remuneration and antibiotic prescriptions. An analysis of patient switches using nationwide registry data

**DOI:** 10.1080/02813432.2026.2715519

**Published:** 2026-08-20

**Authors:** Kristian Bandlien Kraft, Eivor Hovde Hoff, Yana Zykova, Sigurd Høye, Arnstein Mykletun, Kristian Amundsen Østby

**Affiliations:** ^a^ Norwegian Institute of Public Health (NIPH), Cluster for Health Services Research, Oslo, Norway; ^b^ Institute of Health and Society, University of Oslo, Oslo, Norway; ^c^ Department of Community Medicine, Unit – The Arctic University of Norway, Tromsø, Norway; ^d^ Office of the Auditor General of Norway (OAGN), Oslo, Norway; ^e^ School of Business and Economics, UiT - The Arctic University of Norway, Tromsø, Norway; ^f^ The Antibiotic Centre for Primary Care, Department of General Practice, Institute of Health and Society, University of Oslo, Oslo, Norway; ^g^ Nordland Hospital Trust, Centre for Work and Mental Health, Bodø, Norway; ^h^ Centre for Population Health, Haukeland University Hospital, Bergen, Norway

**Keywords:** Antibiotics, remuneration, general practice, fee-for-service, health service research

## Abstract

**Background:**

General practitioners (GPs) play a crucial gatekeeping role in limiting inappropriate antibiotic use. Studies have found that activity-based remuneration of GPs, such as fee-for-service (FFS), is associated with increased antibiotic prescribing. The aim of this study is to compare changes in antibiotic use between patients that switch between GPs with different remuneration types.

**Methods:**

We use individual-level nationwide registries from 2008 to 2019. Patients who initiated a switch of GP during this period are included (*N* = 508 088). We apply a difference-in-differences design to compare patients that switch between GPs with different remuneration types to patients that switch between GPs with similar remuneration types (FFS/Capitation versus salary remuneration).

**Findings:**

Patients who switch to an FFS GP experience an increase in antibiotic use, whereas those who switch to a salaried GP show a decrease. Patients switching from a salaried GP to an FFS GP (compared with salaried-to-salaried switchers) experience a monthly increase of 85 defined daily doses (DDD) of antibiotics per 1 000 patients (SD = 20). Conversely, patients switching from an FFS GP to a salaried GP (compared with FFS-to-FFS switchers) reduce their monthly antibiotics use by 21 DDD per 1000 (SD =7).

**Interpretation:**

Our study indicates that GPs’ remuneration type affects patients’ antibiotic use, with FFS/CAP remuneration being associated with increased use of antibiotics. This relationship might be mediated by differences in consultation frequency, and systematic selection of GPs may also contribute to the observed differences.

## Introduction

Antimicrobial resistance (AMR) is among the greatest threats to global health. The use of antibiotics is a key driver for resistance development, and a considerable proportion of prescribed antibiotics is considered inappropriate [1,2]. Therefore, it is crucial to find means to reduce inappropriate antibiotic consumption. General practitioners (GPs) are among the main prescribers of antibiotics in high-income countries [3], and play an important role in gatekeeping antibiotic use.

According to the OECD, Norway had a total antibiotic consumption level of around 16 defined daily doses (DDD) per 1 000 patients per day in 2015 [4]. This number is lower than the OECD average of 23 DDD but is not among the lowest consumption levels. From 2012 to 2020, Norway experienced a decreasing trend in antibiotic prescription rates, however this trend was reversed in 2021 [5]. It is estimated that 84% of antibiotics dispensed to humans are prescribed in a primary care setting [6].

A range of factors may contribute to inappropriate prescribing of antibiotics in primary care, including factors related to clinician attitude, patient expectations, and the practice environment [3]. This paper investigates the relationship between antibiotic prescriptions and the GPs’ remuneration scheme using data from Norwegian registries. Three types of remuneration are relevant in this setting: fee-for-service (FFS), capitation and salary. FFS reimburses GPs for each consultation and procedure performed, encouraging greater activity. Capitation remunerates GPs based on the number of patients they serve, incentivising GPs to expand their patient lists. In contrast, salaried GPs receive a fixed wage not directly dependent on their activity or number of patients.

Previous research has found a relationship between type of remuneration and the volume of antibiotics prescribed. Two studies found that FFS remuneration, compared to salary, is associated with higher antibiotic prescribing [7,8], while a third did not observe such an association [9]. A recent paper found that GPs prescribe more antibiotics when being remunerated by FFS and capitation, as compared to fixed salary [10]. The relationship between GPs’ remuneration type and antibiotic prescribing can be linked through GPs’ response to financial incentives and the way they organise their practice. In particular, studies from other settings suggest that stronger financial incentives to prescribe tend to increase prescribing levels, whereas stronger incentives not to prescribe tend to reduce them [11–13]. Also, GPs often adjust their practice style in response to new financial incentives or changes in remuneration schemes [11,14–16]. Furthermore, studies have emphasised factors such as consultation duration, the number of listed patients, and the volume of consultations as relevant for antibiotic prescription levels [3,8^,^^9^,^17–25^,^30^]. A Norwegian preprint study shows that GPs paid by FFS and capitation have a higher volume of consultations per patient and shorter mean consultation duration than salaried GPs [26]. Therefore, it is important to understand both the relationship between GPs’ remuneration and practice style, and the relationship between practice style and antibiotics prescription levels.

There are several associational studies in the literature, and one study examines GPs that switch between remuneration schemes [7–10]. This paper’s contribution to the literature is that we follow the same patients as they switch between different GPs, using rich nationwide data spanning several years. We apply a difference-in-differences (DiD) design that compares changes in the antibiotic prescription levels of patients switching between GPs with different remuneration schemes to a comparable group of patients. This design accounts for time-invariant patient confounders and contributes in the effort to better understand the relationship between GP remuneration and antibiotic prescriptions. In this paper, we compare patients switching between GPs with different remuneration to patients switching between GPs with similar remuneration.

## Institutional background

In Norway, most GPs (approximately 85% to 95% during our study period) are self-employed and remunerated by a combination of FFS and capitation (from now on referred to as FFS GPs). The FFS component constitutes approximately 70% of the earnings for self-employed GPs, while around 30% comes from capitation. The remaining GPs are employed by municipalities and are mainly remunerated by salary, although some receive a percentage of FFS earnings. FFS GPs are older and consist of relatively more male physicians. There are also differences in the schemes’ urban-rural distribution, with salary being more common in rural areas.

Municipalities are responsible for the provision of primary care services, including GP services. All Norwegian residents have the right to be listed with a specific regular GP and can switch GP twice a year. Reasons for switching GP may include dissatisfaction, relocation, or other preferences. Patients’ awareness of GPs’ type of remuneration is generally limited. Patients may visit out-of-hours (OOH) services for acute medical care, and around 80% of GPs also work in an OOH clinic [27]. GPs serve a gatekeeping function for several health services and welfare benefits, including prescribing of medications, referrals to specialist services, and sick leave certifications.

## Materials and methods

### Data sources

We used comprehensive nationwide registry data from several sources for the period from January 1, 2008, to December 31, 2019. The data have been merged using a pseudonymized personal identification id for all patients and GPs.

*The Norwegian General Practitioner Registry* (FLO) provided data on GP-patient linkages (including reason for switching), the GPs (age, sex, remuneration scheme), and the patients (age and sex). The *Norwegian Prescribed Drug Registry* (NorPD) contains data on all antibiotics dispensed from Norwegian pharmacies. This dataset does not include prescriptions that have not been collected at pharmacies or antibiotics used in institutions (e.g. hospitals or nursing homes). *Statistics Norway* (SSB) provided sociodemographic variables for patients, including urbanicity, education, and household income. Data on consultations and activity were collected from the *Norwegian Control and Payment of Health Reimbursement Database* (KUHR).

The Norwegian Institute of Public Health conducted a privacy impact assessment regarding the data used, and the Regional Committee for Medical Research Ethics in Southeast Norway granted an exemption from confidentiality (29 October 2020, [#118472]).

### Study sample

Our study sample consists of patients (list patients) who initiated a switch of GP between January 1, 2009, and December 31, 2018 (*N* = 508 088). We observe one GP switch per patient,^1^ and observe patients monthly for two years prior to the switch (pre-period) and three years following the switch (post-period). All included patients are linked to one GP in the pre-period (pre-GP) and another GP in the post-period (post-GP). Patients not listed with either GP for the whole observation period were excluded.^2^ We only included switches where both the pre- and post-GP were actively treating their list patients for at least 10 out of 12 months prior to (pre-GPs) or following (post-GPs) the switch. A GP was considered actively treating list patients during a given month if they conducted at least 15% of their list patients’ contacts. Since month −1 is when most switches occur, this month does not clearly belong to either the pre- or post-period. We include this month in analyses conducted on monthly level but exclude it from Table 2. In addition, we conducted sensitivity analyses to assess the robustness of the results. In one sensitivity analysis, we removed relative month −1 (Supplementary Table 6), and in another we characterize it as the post-period (Supplementary Table 7).

### Variables

#### Outcome variable: antibiotic prescriptions

Our outcome variable is the number of defined daily doses (DDD) of systemic antibiotics (ATC code “J01”) ^3^ dispensed per 1 000 patients per month.^4^ In the main analyses, we do not distinguish between types of prescribers (e.g. the patient’s regular GP, another GP or another prescriber) and include all prescriptions, regardless of who prescribed them. This decision is based on the possibility of systematic differences in patients’ access to their regular GP, which may lead them to seek prescriptions from other providers (e.g. out-of-hours services). We therefore consider the total dispensing of antibiotics to be a more correct measure of the patient’s use. As a sensitivity analysis, we have included an analysis where the outcome is limited to antibiotics only prescribed by the patient’s own GP in the Supplementary Table 1.

#### Exposure: switching remuneration scheme

We categorise patients into four groups based on the possible types of switches between the two main remuneration schemes: FFS and salary. Patients switching between GPs with similar remuneration schemes are placed in our comparison groups, while patients switching between GPs with different remuneration schemes are placed in our exposure groups:

**Table:** 

Switching group	Pre period GP	Post period GP
Exposure Group 1	FFS	Salary
Comparison Group 1	FFS	FFS
Exposure Group 2	Salary	FFS
Comparison Group 2	Salary	Salary

#### Type of prescriber

In some analyses, we distinguish between five types of prescribers. Prescriptions are categorised based on the prescriber ID as follows: If the prescriber ID matches the ID of the patient’s listed GP in the given month, the prescription is classified as from the patient’s (i) *own GP*. We further categorise prescriptions into (ii) *old/new GP,* classifying cases where the prescriber in the pre (post) period was the post (pre) GP. If the antibiotics are prescribed by a GP working at the same office as the regular GP or a GP registered as a locum on the regular GP’s list for the given month, the prescription is classified as from a (iii) *GP at the same office*. If no such matches are found, but the prescriber ID matches another list-holding GP the given month, the prescription is classified as (iv) *another GP*. All other prescriptions are classified as (v) *other prescriber*. This category includes prescriptions from out-of-office services, dentists, private health services, hospitals (only those collected at a pharmacy), or other sources (such as prescriptions to family or friends).

#### Other variables

We also use other demographic variables. *Urbanicity* is measured using the urbanicity of patients’ municipality of residence, based on Statistics Norway’s centrality index, which ranks municipalities from 0 (most rural) to 1000 (most urban). For *education*, we distinguish between high (university/college), medium (upper secondary/vocational), and low (below upper secondary) education. *Household income* is operationalized as quintiles (1 - lowest, 5 - highest) of household income divided by the number of consumption units (equivalized income).

### Analyses

**Figure 1.**
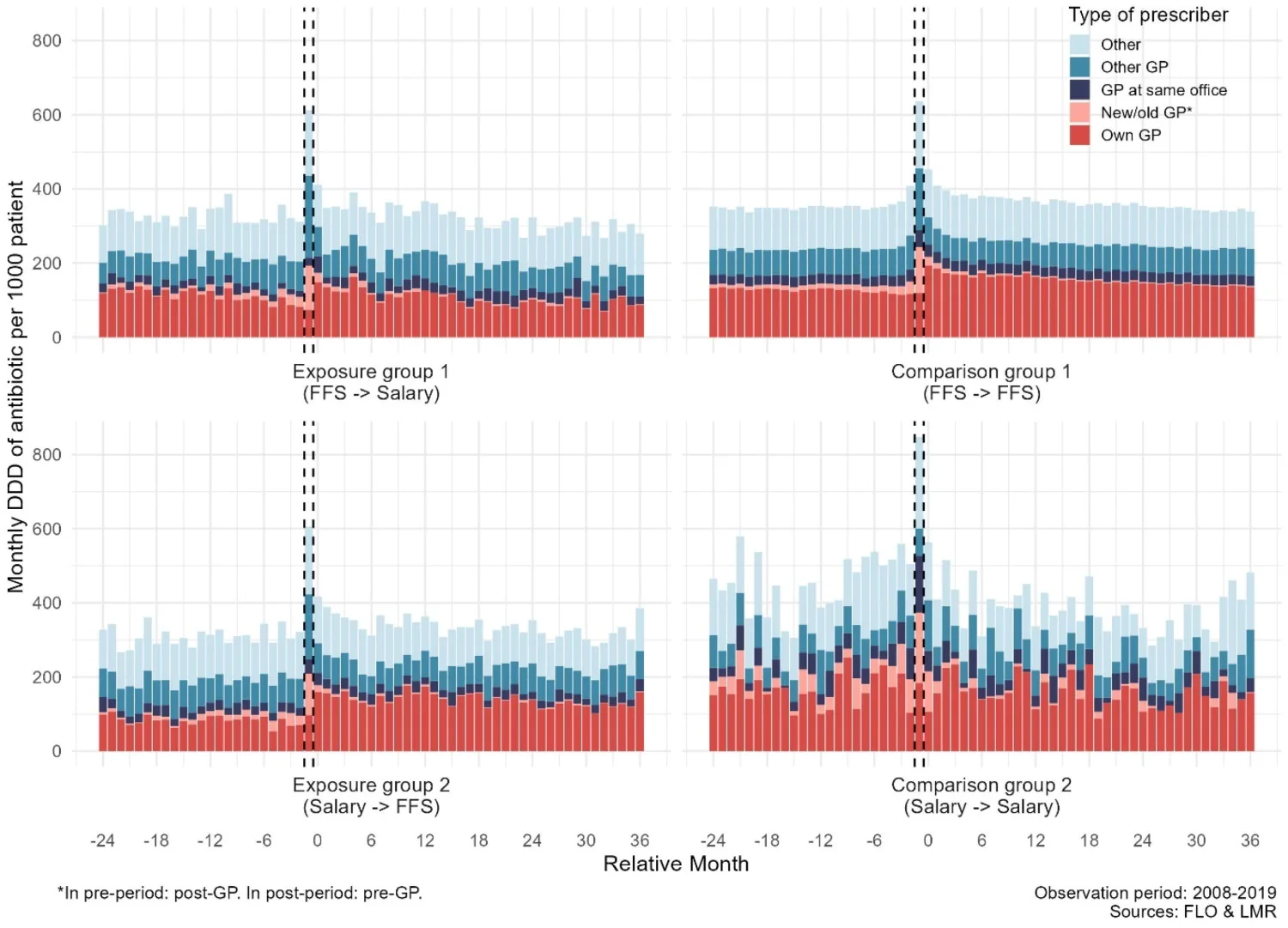
Monthly DDD of antibiotics per 1000 patient by type of prescriber, over each group. Figure notes: −1 represents the switching month and is marked with vertical dashed lines. The spike observed in this month is likely because of an increased need for health services (like antibiotics).

**Figure 2.**
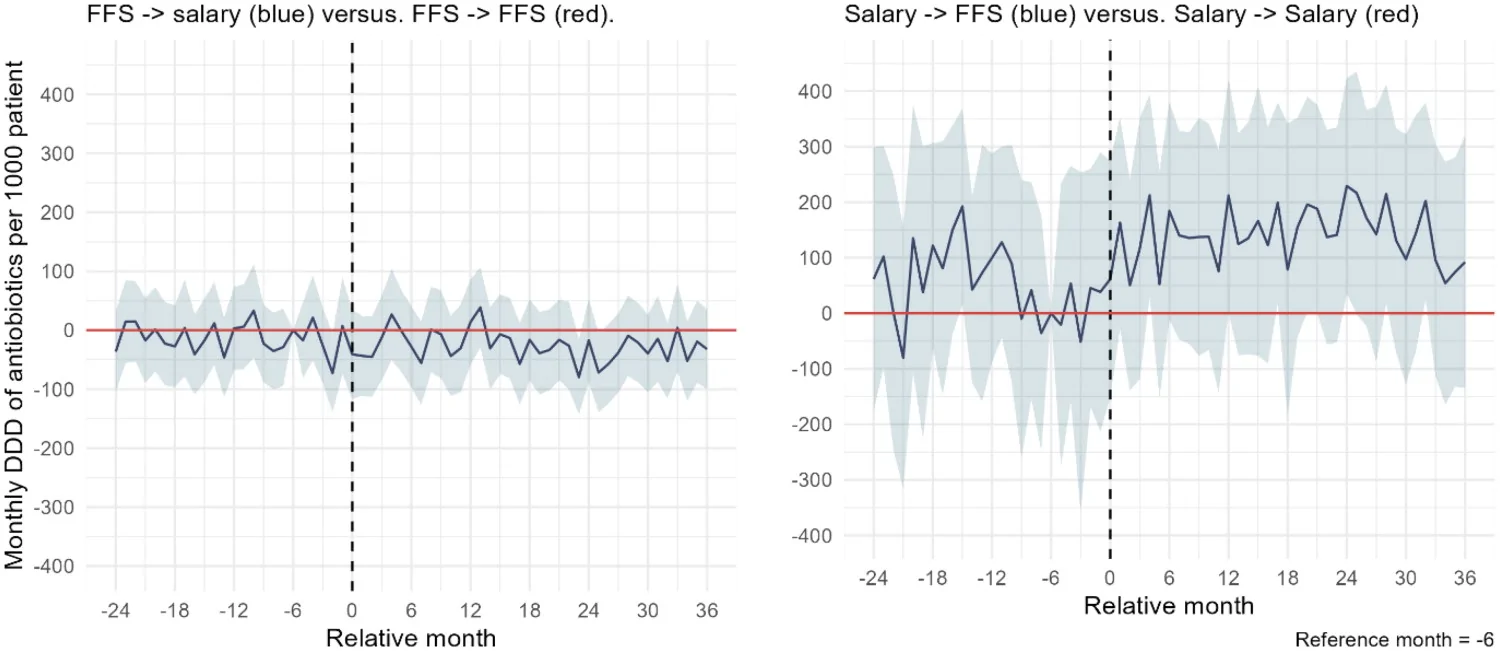
Estimated monthly DDD of antibiotics per 1 000 patients per month. *Notes:* Monthly estimates using monthly estimated difference between exposure and comparison groups. Similar specifications as model 1 and 2 in Table 3.

First, we present descriptive analyses of the groups’ antibiotic use by prescriber type over the observation period (Figure 1 & Table 2). Next, we employ a difference-in-differences (DiD) design on patients that switch to a GP with another remuneration with patients that switch to a GP with the same remuneration. Since patients generally do not have knowledge of the GPs’ remuneration, we argue that the choice of a new GP is not directly influenced by GPs’ remuneration. We first present the event study, where we estimate the difference in antibiotics rate between the exposure- and comparison groups per month (Figure 2).

Furthermore, in Table 3, we conduct a DiD analysis, where we compare the groups’ antibiotic use between the pre- and post-period. We present two models on switching from an FFS GP to a salaried GP (Exposure and Comparison groups 1), and two models for switching from salaried to FFS (Exposure and Comparison groups 2). For each of the analyses, one model shows crude estimates, while the other adjusts for GPs’ age, sex and urbanicity. In all models, we use OLS regression with fixed effects on calendar month and patients, and standard errors are clustered at the GP level. We have not applied recent advancements in the DiD literature because the aim of this study is not to empirically demonstrate causal effects [28]. Rather, the analysis is intended to provide indications that are consistent with several possible interpretations, including a causal one.

We have conducted several supplementary analyses to assess the robustness of our findings exploring support for mechanisms. In some of the sensitivity analyses, we have used different outcome measures: antibiotics prescribed only by the patient’s own GP (Supplementary Table 1) and whether antibiotics were prescribed in a given month (Supplementary Table 2). We have also conducted the analyses on different sub-samples of movers (Supplementary Tables 3 A & B) and non-movers (Supplementary Tables 4 A & B). Movers are defined as patients whose most frequent municipality of residence differs between the pre- and post-periods, whereas non-movers are those whose most frequent municipality remains the same across both periods. We have also estimated alternative model specifications, including matching patients by switching year (Supplementary Table 5); excluding relative month −1 (Supplementary Table 6), redefining relative month −1 as part of the post-period (Supplementary Table 7), and using a broader sample definition (Supplementary Table 8). Last, we have conducted the two sub-analyses on changes in the number of consultations (Supplementary Tables 9 A & B).

## Results

Table 1 presents descriptive statistics for patients in the four switching groups, along with their GPs in the pre- and post-periods. Most GPs are remunerated by FFS, and consequently most switches (*N* = 477 286) occur between one FFS GP and another FFS GP. The other three groups are smaller, consisting of between 2 000 to 17 000 patients. Female patients are slightly overrepresented in all groups, and the highest share of females is found in Comparison Group 2. Mean age varies between 33 and 38 years across the groups. There are notable urban/rural differences. Comparison Group 1 is generally located in more urban areas, while the Comparison Group 2 is predominantly rural.

**Table 1.** Descriptives of groups.

Pre-period GP	FFS				Salary			
Post period GP	Salary (Exposure Group 1)		FFS (Comparison Group 1)		FFS (Exposure Group 2)		Salary (Comparison Group 2)	
Patients^1^								
N	12 171		477 287		16 501		2 129	
% Male	49 %		49 %		50 %		44 %	
Mean age (SD)	33 (19)		35 (20)		33 (19)		38 (22)	
Mean urbanicity (SD)	779 (159)		855 (116)		793 (162)		711 (165)	
% Low education	32 %		40 %		36 %		41 %	
% Medium education	39 %		30 %		27 %		32 %	
% High education	30 %		30 %		37 %		27 %	
Mean household income quintile (SD)	2.82 (1.38)		3.02 (1.42)		2.94 (1.42)		3 (1.38)	
GP characteristics^2^	Pre GP (FFS)	Post GP (Salary)	Pre GP (FFS)	Post GP (FFS)	Pre GP (Salary)	Post GP (FFS)	Pre GP (Salary)	Post GP (Salary)
N Unique GPs	3199	228	4673	4721	265	3109	190	170
% Male GPs	68 %	54 %	69 %	64 %	59 %	62 %	64 %	66 %
Mean age GPs (SD)	51 (10)	49 (11)	51 (10)	47 (10)	53 (10)	47 (10)	52 (9)	50 (10)
Mean urbanicity^3^ (SD)	783 (153)	776 (168)	854 (117)	856 (114)	782 (166)	812 (153)	710 (163)	714 (167)

1: Patient characteristics are measured in month 0. 2: GP characteristics are calculated by weighting each GP according to the number of patient-months registered with them (except N Unique GPs).

The sample consists of a larger number of unique FFS GPs than salaried GPs, consistent across all groups. There is a higher share of male GPs in the pre-period for Exposure and Comparison group 1, while a more even share between the periods for Exposure and Comparison group 2. Pre-period GPs are slightly older than post-period GPs across all groups. Minor changes in urbanicity are observed from the pre- and post-periods for the exposure groups.

Figure 1 shows monthly DDD of antibiotics in the pre- and post-periods by switching groups and type of prescriber. Patients that switch to an FFS GP (Comparison Group 1 & Exposure Group 2) experience a minor upward shift that coincides with the GP switch. Although they experience a weak downward trend in the post-period, this results in an increase in mean monthly DDD per 1 000 patients from 306 to 335 for Exposure Group 2, and 348 to 363 for Comparison Group 1 (FFS to FFS) (Table 2). Patients switching to a salaried GP do not experience a similar increase. Patients in Exposure Group 1 experience a marginal reduction from 323 to 321 DDD of antibiotics per month per 1 000 patients, while the similar number for Comparison Group 2 is 434 to 381.

Two main types of prescribers account for approximately one third of all antibiotics each: the patients’ own GP and another prescriber who is not a GP in the given month (Figure 1). The remaining antibiotics are prescribed by another GP (both the old/new GP, GPs at the same office, or other GPs). Among patients who switch to a GP with a different remuneration scheme, both the number and proportion of antibiotics prescribed by their own GP are higher when the GP is remunerated through FFS compared with a salaried GP (Table 2).

**Table 2.** Antibiotics and consultations in pre- and post-periods by groups.

Group	FFS -> Salary (Exposure Group 1)		FFS -> FFS (Comparison Group 1)		Salary -> FFS (Exposure Group 2)		Salary -> Salary (Comparison Group 2)	
Monthly per 1 000 patients	Pre-period (FFS)	Post-period (Salary)	Pre-period (FFS)	Post-period (FFS)	Pre-period (Salary)	Post-period (FFS)	Pre-period (Salary)	Post-period (Salary)
Antibiotics								
DDD per 1 000 patients	323	321	348	363	306	335	434	381
Own GP (share of total)	114 (35 %)	106 (33 %)	127 (36 %)	155 (43 %)	83 (27 %)	139 (41 %)	165 (38 %)	156 (41 %)
Old/new GP (share of total)	13 (4 %)	5 (2 %)	14 (4 %)	5 (1 %)	13 (4 %)	4 (1 %)	39 (9 %)	14 (4 %)
GPs at the same office (share of total)	21 (7 %)	27 (9 %)	27 (8 %)	28 (8 %)	25 (8 %)	25 (8 %)	43 (10 %)	39 (10 %)
Another GP (share of total)	66 (21 %)	68 (21 %)	71 (20 %)	66 (18 %)	71 (23 %)	65 (19 %)	67 (15 %)	69 (18 %)
Other (share of total)	112 (35 %)	116 (36 %)	115 (33 %)	111 (31 %)	118 (39 %)	103 (31 %)	143 (33 %)	112 (29 %)

*Notes:* Antibiotics in the month prior to the switch are excluded.

Figure 2 shows the results from the event study where we estimate the antibiotics rate per month. In the left panel, patients switching from an FFS GP to a salaried GP (Exposure Group 1, blue line) are compared to patients switching from an FFS GP to another FFS GP (Comparison Group 1, red line). The Exposure group 1 seems to experience a slight downward trend in the pre-period relative to the comparison group. Further, there are seemingly no significant changes in monthly antibiotic levels throughout the post period.

In the right panel, patients switching from a salaried GP to an FFS GP (Exposure Group 2, blue line) are compared with patients who switch from a salaried GP to another salaried GP (Comparison Group 2, red line). The pre-period is showing greater fluctuation than the left panel, likely due to the smaller number of patients in the Comparison Group. The Exposure Group displays an increase followed by a decline in the pre-period relative to the Comparison Group, although the confidence intervals overlap in all but one month. This pre-trend introduces some uncertainty regarding a causal interpretation. In the post-period, the Exposure Group shows a moderate and sustained increase in antibiotic use compared with the Comparison Group. However, most estimates are not statistically significant at the monthly level.

The DiD estimates are presented in Table 3. In the crude models, switching from an FFS GP to a salaried GP reduces monthly DDD per 1 000 patients by −19, compared to switching between two FFS GPs (Model 1). Conversely, switching from a salaried GP to an FFS GP, compared to switching between two salaried GPs, results in an increase of 88 DDD of antibiotics per 1 000 patients (Model 2). When adjusting for the sex and age of the GP, in addition to urbanicity, the results remain similar, with only marginal changes (Model 3 & 4). The first estimate changes from −19 to −21, while the second changes from 88 to 85.

**Table 3.** DiD Regression results: monthly DDD of antibiotics per 1 000 patients.

Outcome:	Monthly DDD of antibiotics per 1 000 patients			
Specification	Crude		Adjusted	
Models:	Model 1	Model 2	Model 3	Model 4
Switch from (pre-period):	FFS	Salary	FFS	Salary
Post*FFS		87.91***		84.64***
		(20.09)		(19.56)
Post*Salary	−19.35**		−21.27**	
	(7.328)		(7.327)	
Patient FE	Yes	Yes	Yes	Yes
Calendar month FE	Yes	Yes	Yes	Yes
Adjustment variables^1^	No	No	Yes	Yes
GP clustered SE	Yes	Yes	Yes	Yes
R2	0.07704	0.07553	0.07706	0.07525
Observations	29 856 938	1 136 430	29 784 866	1 133 595

*Notes:* DiD estimates using ordinary least square regression standard errors in parentheses. Significance codes: *** = 0.001, ** = 0.01, * = 0.05 1: GP’s sex, GP’s age, and urbanicity.

We have conducted several analyses as described in methods section and presented in the supplementary file. In general, most of the sensitivity analyses are consistent with the overall findings. However, some of the estimates in the movers and non-movers’ analyses become weaker and statistically insignificant (Supplementary Tables 3 A, 3B, 4 A and 4B). In the subsample of movers, this occurs for the analyses of patients who start with a salaried GP, whereas the opposite pattern is observed in the non-movers subsample. Nonetheless, most of these estimates lie close to conventional significance thresholds, and the direction of the associations remains unchanged.

We have also conducted two sub-analyses on the relation between remuneration and consultation frequency (Supplementary Tables 7 A & B). In both analyses, we find that patients receive a higher number of consultations when switching to an FFS GP, as compared to a salaried GP.

## Discussion

Our study finds that patients who switch to an FFS GP experience an increase in antibiotic use, whereas those who switch to a salaried GP show a decrease. Patients switching from a salaried to an FFS GP (as compared to salaried to salaried) experience an increase of 85 DDD of antibiotics per 1 000 patients (SD = 20). Patients switching from an FFS GP to a salaried GP (as compared to FFS to FFS) yield a reduction of 19 DDD per 1 000 patients (SD = 7). Among patients who switch between remuneration schemes, a larger share of antibiotics is prescribed by their own GP when their GP is remunerated through FFS compared with salary. The sub-analyses show that patients receive a higher number of consultations when switching to an FFS GP, as compared to a salaried GP.

Our results concur with most of the previous research on GP remuneration and antibiotics [7–10] that find that patients of FFS GPs receive more antibiotics. There are at least two, not mutually exclusive, interpretations of the main results [1]: a causal interpretation attributed to remuneration type, and [2] systematic differences between GPs with different remuneration types. This study cannot determine the relative importance of these interpretations, but we discuss these interpretations in the following paragraphs.

First, the sample of patient-initiated switches included in this study does not allow us to empirically demonstrate clear causal effects. Additionally, both the less stable pre-trend and the sensitivity analyses of movers introduce some uncertainty to this interpretation. However, our study cannot exclude a possible causal relationship, and we argue that there are indications that support the theoretical mechanism of consultation frequency in explaining why FFS remuneration may cause GPs to prescribe more. FFS is designed to stimulate activity, and FFS GPs conduct more consultations per patient compared to salaried GPs. A higher consultation rate driven by other causes than morbidity, such as greater availability, may lead to increased antibiotic consumption [21]. Since antibiotic prescription decisions might not always be clear-cut, a higher consultation rate may increase both appropriate and inappropriate prescribing of antibiotics. Our results provide some support for this mechanism. While all groups experience an increased consultation rate in the post period, patients switching to an FFS GP show a larger increase compared to those switching to a salaried GP (Supplementary Tables 7 A & B). This indicates that the level of antibiotic prescriptions can be partly driven by consultation frequency, with higher levels among FFS GPs, potentially reflecting a greater degree of supplier-induced demand [29].

Another indication of this mechanism is that FFS GPs account for a larger share of the antibiotics prescribed to their patients compared to salaried GPs (Figure 1), suggesting greater availability and continuity of care. While these may be considered as markers of quality, they may also contribute to higher prescribing, since the prescribing behavior of the patient’s own GPs drives the overall prescription rates for FFS GPs. This highlights a potential tension between competing quality indicators: reducing antibiotic use on the one hand and ensuring availability and continuity of care on the other. This should be further examined in future research, in addition to other treatments and welfare benefits which may follow a similar logic, such as prescribing addictive drugs, referrals to specialist care or issuing sickness certifications.

Second, GPs with different remuneration schemes may systematically differ. For instance, there is an urban-rural difference between FFS and salaried GPs. Therefore, switching GP due to relocation might also mean greater or poorer access to GPs and other healthcare services. To investigate this, we conducted a subsample analysis including only patients who did not relocate. Results show that this weakens the analysis of patients starting with an FFS GP. Nevertheless, systematic differences in the types of GPs recruited into different schemes may also represent an indirect contributing mechanism. If more liberal prescribers are more drawn to FFS remuneration, systematic selection based on prescribing attitudes could contribute to the observed differences. Our results neither favour nor disfavour such an interpretation, and we encourage further research on this question.

A strength of this paper is the use of comprehensive nationwide registry data spanning ten years, providing detailed and high-quality material. Further, the DID design effectively minimises the influence of patient invariant confounding in the relationship between remuneration and antibiotic prescriptions. However, we cannot rule out the possibility that GP confounding, time-variant patient confounding or different underlying reasons for switching can contribute to explaining the results. We study patient-initiated switches, and the results may not directly translate to other settings. These patients show a marked increase in antibiotic use around time of the switch, which could reflect either a health event triggering the switch or the switch itself increasing patients’ propensity to visit their new GP. Although this has implications for generalisability, our sensitivity analyses show that the results are robust to different interpretations of this spike, i.e. the placement of the cut-off between pre- and post-periods.

There is an asymmetry in the findings: we find a stronger increase in switching from a salaried GP to an FFS GP, whereas the results are weaker the other way around. Although one would intuitively expect symmetry, this may still have reasonable explanations. For example, since patient expectations are an important driver of antibiotic use, one explanation may be that it is harder for a GP to reduce antibiotic use than to increase it.

Our study provides indications that GPs’ remuneration plays a role for antibiotic prescribing. An estimate of 85 (21) DDD of antibiotics per 1 000 patients per month corresponds to 1020 (252) DDD yearly per 1 000 patients, equating to 17.5 (4.3) % of the 2015 level [4]. However, these numbers cannot be interpreted as the effect of a transition between remuneration schemes, as we do not observe clear changes in both switching directions, unobserved confounders may be present, and the transition itself may introduce new factors. Nevertheless, how GPs are remunerated may be one of the factors to consider in the efforts to reduce antibiotic consumption.

## Conclusion

This study finds that patients switching from a salaried GP to an FFS GP experience a larger increase in antibiotic use compared to salaried-to-salaried switchers. Conversely, patients switching from an FFS GP to a salaried GP exhibit a modest reduction in antibiotic use relative to FFS-to-FFS switchers. Both the type of remuneration and GP selection may contribute to the findings. FFS remuneration may influence the GPs’ antibiotic prescribing by increasing the frequency of consultations. The evidence leaves some uncertainty regarding a clear causal interpretation, and systematic selection of GPs may also contribute to the observed differences.

## Supplementary Material

Supplemental Material

## Data Availability

The data that support the findings of this study are available from *Helsedataservice* and *Statistics Norway*. Restrictions apply to the availability of these data, which were used under license for this study.

## Artificial intelligence

During the preparation of this work the authors used GPT-4o mini and Claude Sonnet 5 in order to improve language. After using these tools/services, the authors reviewed and edited the content as needed and take full responsibility for the content of the published article.

## Notes

1 If a patient switched GP several times between 2009-2018, we used the first switch.

2 In a sensitivity analysis (Supplementary Table 8), we also included patients that were listed to each GP for at least one year. Patients were censored at the time of leaving the GP’s list.

3 We excluded methenamine (‘J01X X05’), as it is a preventive antiseptic not regarded to cause antimicrobial resistance.

4 We have included a sensitivity analysis where we use the at least one prescription of antibiotics prescribed in supplementary table 2.
