# Supplementary material for: General practitioners’ type of remuneration and antibiotic prescriptions. An analysis of patient switches using nationwide registry data
Source: Scand J Prim Health Care. 2026 Aug 20;44(1):2715519. doi: 10.1080/02813432.2026.2715519 (PMC13495028; doi:10.1080/02813432.2026.2715519)
Supplement: Supplementary file 1 [file IPRI_A_2715519_SM1833.docx]

# Supplementary

## Supplementary table 1: DiD regression results: Sensitivity analysis of antibiotic prescriptions only prescribed by own GP

| Outcome: | Defined daily doses of antibiotics by own GP per 1000 patients | | | |  |
| --- | --- | --- | --- | --- | --- |
| Specification | Crude | | Adjusted | |  |
| Models: | Model 1 | Model 2 | Model 3 | Model 4 |  |
| Switch from (pre-period): | FFS | Salary | FFS | Salary |  |
|  |  |  |  |  |  |
| Post*FFS |  | 64.47*** |  | 64.93*** |  |
|  |  | (17.42) |  | (16.61) |  |
| Post*Salary | -35.25*** |  | -36.87*** |  |  |
|  | (5.078) |  | (5.040) |  |  |
| Patient FE | Yes | Yes | Yes | Yes |  |
| Calender month FE | Yes | Yes | Yes | Yes |  |
| Adjustment variables^1^ | No | No | Yes | Yes |  |
| GP clustered SE | Yes | Yes | Yes | Yes |  |
| R2 | 0.05852 | 0.06165 | 0.05857 | 0.06173 |  |
| Observations | 29,856,938 | 1,136,430 | 29,784,866 | 1,133,595 |  |

Table notes: . DiD estimates using ordinary least square regression 95 % confidence intervals in parentheses. Significance codes: *** = 0.001, ** = 0.01, * = 0.05 1: GP’s sex, GP’s age, and urbanicity

## Supplementary table 2: DiD regression results: Sensitivity analysis of alternative outcome measure: at least one antibiotic prescription the given month

| Outcome: | Antibiotic prescriptions per 1000 patients | | | |  |
| --- | --- | --- | --- | --- | --- |
| Specification | Crude | | Adjusted | |  |
| Models: | Model 1 | Model 2 | Model 3 | Model 4 |  |
| Switch from (pre-period): | FFS | Salary | FFS | Salary |  |
|  |  |  |  |  |  |
| Post*FFS |  | 4.591*** |  | 4.373*** |  |
|  |  | (0.8931) |  | (0.8882) |  |
| Post*Salary | -1.237** |  | -1.320*** |  |  |
|  | (0.3897) |  | (0.3890) |  |  |
| Patient FE | Yes | Yes | Yes | Yes |  |
| Relative month FE | Yes | Yes | Yes | Yes |  |
| Year FE | Yes | Yes | Yes | Yes |  |
| Adjustment variables^1^ | No | No | Yes | Yes |  |
| GP clustered SE | Yes | Yes | Yes | Yes |  |
| R2 | 0.07271 | 0.07404 | 0.07291 | 0.07417 |  |
| Observations | 29,856,938 | 1,136,430 | 29,784,866 | 1,133,595 |  |

Table notes: . DiD estimates using ordinary least square regression 95 % confidence intervals in parentheses. Significance codes: *** = 0.001, ** = 0.01, * = 0.05 1: GP’s sex, GP’s age, and urbanicity

## Supplementary table 3A & B: DiD regression results: Sensitivity analysis of subsample of movers

Supplementary table 3A: Defined daily doses of antibiotics per 1000 patients

| Outcome: | Defined daily doses of antibiotics per 1000 patients | | | |  |
| --- | --- | --- | --- | --- | --- |
| Specification | Crude | | Adjusted | |  |
| Models: | Model 1 | Model 2 | Model 3 | Model 4 |  |
| Switch from (pre-period): | FFS | Salary | FFS | Salary |  |
|  |  |  |  |  |  |
| Post*FFS |  | 39.43 |  | 34.16 |  |
|  |  | (38.16) |  | (37.95) |  |
| Post*Salary | -17.81* |  | -19.55* |  |  |
|  | (8.784) |  | (8.780) |  |  |
| Patient FE | Yes | Yes | Yes | Yes |  |
| Calender month FE | Yes | Yes | Yes | Yes |  |
| Adjustment variables^1^ | No | No | Yes | Yes |  |
| GP clustered SE | Yes | Yes | Yes | Yes |  |
| R2 | 0.07256 | 0.08812 | 0.07260 | 0.08815 |  |
| Observations | 14,045,738 | 533,140 | 14,002,684 | 531,121 |  |

Table notes: . DiD estimates using ordinary least square regression 95 % confidence intervals in parentheses. Significance codes: *** = 0.001, ** = 0.01, * = 0.05 1: GP’s sex, GP’s age, and urbanicity

Supplementary table 3B: At least one antibiotic prescription the given month per 1000 patients

| Outcome: | Antibiotic prescriptions per 1000 patients | | | |  |
| --- | --- | --- | --- | --- | --- |
| Specification | Crude | | Adjusted | |  |
| Models: | Model 1 | Model 2 | Model 3 | Model 4 |  |
| Switch from (pre-period): | FFS | Salary | FFS | Salary |  |
|  |  |  |  |  |  |
| Post*FFS |  | 3.636* |  | 3.284 |  |
|  |  | (1.681) |  | (1.681) |  |
| Post*Salary | -1.710*** |  | -1.726*** |  |  |
|  | (0.4512) |  | (0.4517) |  |  |
| Patient FE | Yes | Yes | Yes | Yes |  |
| Calender month FE | Yes | Yes | Yes | Yes |  |
| Adjustment variables^1^ | No | No | Yes | Yes |  |
| GP clustered SE | Yes | Yes | Yes | Yes |  |
| R2 | 0.05805 | 0.06319 | 0.05810 | 0.06327 |  |
| Observations | 14,045,738 | 533,140 | 14,002,684 | 531,121 |  |

Table notes: . DiD estimates using ordinary least square regression 95 % confidence intervals in parentheses. Significance codes: *** = 0.001, ** = 0.01, * = 0.05 1: GP’s sex, GP’s age, and urbanicity

## Supplementary table 4A & B: DiD regression results: Sensitivity analysis of subsample of non-movers

Supplementary table 4A: Defined daily doses of antibiotics per 1000 patients

| Outcome: | Defined daily doses of antibiotics per 1000 patients | | | |  |
| --- | --- | --- | --- | --- | --- |
| Specification | Crude | | Adjusted | |  |
| Models: | Model 1 | Model 2 | Model 3 | Model 4 |  |
| Switch from (pre-period): | FFS | Salary | FFS | Salary |  |
|  |  |  |  |  |  |
| Post*FFS |  | 91.65*** |  | 93.72*** |  |
|  |  | (24.06) |  | (22.90) |  |
| Post*Salary | -20.96 |  | -23.11 |  |  |
|  | (11.91) |  | (11.93) |  |  |
| Patient FE | Yes | Yes | Yes | Yes |  |
| Calender month FE | Yes | Yes | Yes | Yes |  |
| Adjustment variables^1^ | No | No | Yes | Yes |  |
| GP clustered SE | Yes | Yes | Yes | Yes |  |
| R2 | 0.07256 | 0.08812 | 0.07260 | 0.08815 |  |
| Observations | 15,811,200 | 603,290 | 15,782,182 | 602,474 |  |

Table notes: . DiD estimates using ordinary least square regression 95 % confidence intervals in parentheses. Significance codes: *** = 0.001, ** = 0.01, * = 0.05 1: GP’s sex, GP’s age, and urbanicity

Supplementary table 4B: At least one antibiotic prescription the given month per 1000 patients

| Outcome: | Antibiotic prescriptions per 1000 patients | | | |  |
| --- | --- | --- | --- | --- | --- |
| Specification | Crude | | Adjusted | |  |
| Models: | Model 1 | Model 2 | Model 3 | Model 4 |  |
| Switch from (pre-period): | FFS | Salary | FFS | Salary |  |
|  |  |  |  |  |  |
| Post*FFS |  | 4.417*** |  | 4.582*** |  |
|  |  | (1.148) |  | (1.103) |  |
| Post*Salary | -0.4550 |  | -0.6067 |  |  |
|  | (0.6868) |  | (0.6823) |  |  |
| Patient FE | Yes | Yes | Yes | Yes |  |
| Calender month FE | Yes | Yes | Yes | Yes |  |
| Adjustment variables^1^ | No | No | Yes | Yes |  |
| GP clustered SE | Yes | Yes | Yes | Yes |  |
| R2 | 0.06574 | 0.06469 | 0.06576 | 0.06476 |  |
| Observations | 15,811,200 | 603,290 | 15,782,182 | 602,474 |  |

Table notes: . DiD estimates using ordinary least square regression 95 % confidence intervals in parentheses. Significance codes: *** = 0.001, ** = 0.01, * = 0.05 1: GP’s sex, GP’s age, and urbanicity

## Supplementary table 5: DiD regression results: Sensitivity analysis of matching patient by switching year

| Outcome: | Defined daily doses of antibiotics per 1000 patients | | | |  |
| --- | --- | --- | --- | --- | --- |
| Specification | Crude | | Adjusted | |  |
| Models: | Model 1 | Model 2 | Model 3 | Model 4 |  |
| Switch from (pre-period): | FFS | Salary | FFS | Salary |  |
|  |  |  |  |  |  |
| Post*FFS |  | 71.97*** |  | 68.63*** |  |
|  |  | (24.06) |  | (17.74) |  |
| Post*Salary | -16.51 |  | -19.79* |  |  |
|  | (9.147) |  | (9.130) |  |  |
| Patient FE | Yes | Yes | Yes | Yes |  |
| Calender month FE | Yes | Yes | Yes | Yes |  |
| Adjustment variables^1^ | No | No | Yes | Yes |  |
| GP clustered SE | Yes | Yes | Yes | Yes |  |
| R2 | 0.08745 | 0.07022 | 0.08748 | 0.07007 |  |
| Observations | 2,921,040 | 3,960,240 | 2,914,127 | 3,953,311 |  |

Table notes: . DiD estimates using ordinary least square regression 95 % confidence intervals in parentheses. Significance codes: *** = 0.001, ** = 0.01, * = 0.05 1: GP’s sex, GP’s age, and urbanicity

## Supplementary table 6: DiD regression results: Sensitivity analysis of removing relative month -1

| Outcome: | Defined daily doses of antibiotics per 1000 patients | | | |  |
| --- | --- | --- | --- | --- | --- |
| Specification | Crude | | Adjusted | |  |
| Models: | Model 1 | Model 2 | Model 3 | Model 4 |  |
| Switch from (pre-period): | FFS | Salary | FFS | Salary |  |
|  |  |  |  |  |  |
| Post*FFS |  | 86.52*** |  | 83.91*** |  |
|  |  | (20.15) |  | (19.59) |  |
| Post*Salary | -18.68* |  | -20.48** |  |  |
|  | (7.320) |  | (7.321) |  |  |
| Patient FE | Yes | Yes | Yes | Yes |  |
| Calender month FE | Yes | Yes | Yes | Yes |  |
| Adjustment variables^1^ | No | No | Yes | Yes |  |
| GP clustered SE | Yes | Yes | Yes | Yes |  |
| R2 | 0.07755 | 0.07568 | 0.07758 | 0.07539 |  |
| Observations | 29,367,480 | 1,117,800 | 29,296,080 | 1,114,996 |  |

Table notes: . DiD estimates using ordinary least square regression 95 % confidence intervals in parentheses. Significance codes: *** = 0.001, ** = 0.01, * = 0.05 1: GP’s sex, GP’s age, and urbanicity

## Supplementary table 7: DiD regression results: Sensitivity analysis of defining relative month -1 as part of the post period.

| Outcome: | Defined daily doses of antibiotics per 1000 patients | | | |  |
| --- | --- | --- | --- | --- | --- |
| Specification | Crude | | Adjusted | |  |
| Models: | Model 1 | Model 2 | Model 3 | Model 4 |  |
| Switch from (pre-period): | FFS | Salary | FFS | Salary |  |
|  |  |  |  |  |  |
| Post*FFS |  | 83.94*** |  | 83.11*** |  |
|  |  | (20.46) |  | (19.88) |  |
| Post*Salary | -17.78* |  | -21.01** |  |  |
|  | (7.403) |  | (7.386) |  |  |
| Patient FE | Yes | Yes | Yes | Yes |  |
| Calender month FE | Yes | Yes | Yes | Yes |  |
| Adjustment variables^1^ | No | No | Yes | Yes |  |
| GP clustered SE | Yes | Yes | Yes | Yes |  |
| R2 | 0.07708 | 0.07558 | 0.07711 | 0.07530 |  |
| Observations | 29,856,938 | 1,136,430 | 29,784,866 | 1,133,595 |  |

Table notes: . DiD estimates using ordinary least square regression 95 % confidence intervals in parentheses. Significance codes: *** = 0.001, ** = 0.01, * = 0.05 1: GP’s sex, GP’s age, and urbanicity

## Supplementary table 8: DiD regression results: Sensitivity analysis of broader sample definition

Definition: All patients that were listed to each GP for at least one year. Patients were censored at the time of leaving the GP’s list.

| Outcome: | Defined daily doses of antibiotics per 1000 patients | | | |  |
| --- | --- | --- | --- | --- | --- |
| Specification | Crude | | Adjusted | |  |
| Models: | Model 1 | Model 2 | Model 3 | Model 4 |  |
| Switch from (pre-period): | FFS | Salary | FFS | Salary |  |
|  |  |  |  |  |  |
| Post*FFS |  | 45.67** |  | 43.93** |  |
|  |  | (14.06) |  | (13.87) |  |
| Post*Salary | -16.30** |  | -17.53*** |  |  |
|  | (5.110) |  | (5.133) |  |  |
| Patient FE | Yes | Yes | Yes | Yes |  |
| Calender month FE | Yes | Yes | Yes | Yes |  |
| Adjustment variables^1^ | No | No | Yes | Yes |  |
| GP clustered SE | Yes | Yes | Yes | Yes |  |
| R2 | 0.07708 | 0.07558 | 0.07711 | 0.07530 |  |
| Observations | 53,949,726 | 2,109,285 | 53,597,194 | 2,096,069 |  |

Table notes: . DiD estimates using ordinary least square regression 95 % confidence intervals in parentheses. Significance codes: *** = 0.001, ** = 0.01, * = 0.05 1: GP’s sex, GP’s age, and urbanicity

## Supplementary table 9A & B: DiD regression results: Monthly consultations per 1000 patients

Supplementary table 9A: DiD results of Number of consultations GP per 1000 patients

| Outcome: | Number of consultations GP per 1000 patients | | | |  |
| --- | --- | --- | --- | --- | --- |
| Specification | Crude | | Adjusted | |  |
| Models: | Model 1 | Model 2 | Model 3 | Model 4 |  |
| Switch from (pre-period): | FFS | Salary | FFS | Salary |  |
|  |  |  |  |  |  |
| Post*FFS |  | 31.49*** |  | 29.79*** |  |
|  |  | (3.087) |  | (3.059) |  |
| Post*Salary | -13.66*** |  | -12.77*** |  |  |
|  | (1.536) |  | (1.453) |  |  |
| Patient FE | Yes | Yes | Yes | Yes |  |
| Calender month FE | Yes | Yes | Yes | Yes |  |
| Adjustment variables^1^ | No | No | Yes | Yes |  |
| GP clustered SE | Yes | Yes | Yes | Yes |  |
| R2 | 0.16868 | 0.16077 | 0.16888 | 0.16092 |  |
| Observations | 29,856,938 | 1,136,430 | 29,784,866 | 1,133,595 |  |

Table notes: . DiD estimates using ordinary least square regression 95 % confidence intervals in parentheses. Significance codes: *** = 0.001, ** = 0.01, * = 0.05 1: GP’s sex, GP’s age, and urbanicity

Supplementary table 9B: Descriptive results in pre- and post-periods for the switching groups

| Group | FFS -> Salary  (Exposure Group 1) | | FFS -> FFS  (Comparison Group 1) | | Salary -> FFS  (Exposure Group 2) | | Salary -> Salary  (Comparison Group 2) | |
| --- | --- | --- | --- | --- | --- | --- | --- | --- |
| Monthly per 1 000 patients | Pre-period (FFS) | Post-period (Salary) | Pre-period (FFS) | Post-period (FFS) | Pre-period (Salary) | Post-period (FFS) | Pre-period (Salary) | Post-period (Salary) |
| Consultations  (% increase from pre-period) | 128 | 161  (26 %) | 150 | 195  (30 %) | 124 | 179  (44 %) | 175 | 202  (15 %) |
| DDD of Antibiotics / number of consultations | 2.53 % | 2 % | 2.32 % | 1.86 % | 2.46 % | 1.87 % | 2.48 % | 1.89 % |

Table text: In general, patients visit their GP more frequently in the post-period. Patients listed with FFS GPs have more consultations, and patients switching to FFS GPs experience a greater increase in visit frequency in the post-period compared to patients switching to a salaried GP. Patients switching from a salaried GP to an FFS GP (Exposure Group 2) experience a 44% increase in the number of consultations following the switch, compared to 15 % for patients switching from a salaried to another salaried (Comparison Group 2). For patients switching from an FFS GP to a salaried GP (Exposure Group 1), the number of consultations increase with 26%, compared to 30 % in the Comparison Group 1 (FFS to FFS).
